# Triple Technique for Neglected Locked Patellar Dislocation With Lateral Release, Synthetic Medial Patellofemoral Ligament, and Peroneus Longus Autograft Medial Quadriceps Tendon‐Femoral Ligament Reconstruction

**DOI:** 10.1002/atn2.70243

**Published:** 2026-07-31

**Authors:** Hesham Ahmed Abdul Galil, Moustafa Kamal El Gafary, Amr Samy M. El Hady, Ahmad Hany Khater

**Affiliations:** ^1^ Department of Orthopedic Surgery Nasser Institute for Research and Treatment Cairo Egypt; ^2^ Department of Orthopedic Surgery Capital University (Helwan University) Cairo Egypt; ^3^ Department of Orthopedic Surgery Ain Shams University Cairo Egypt

## Abstract

Patellar dislocation is a frequent emergency presentation. First‐time dislocations are associated with medial patellofemoral ligament injury in up to 94.7% of cases. Although many reduce spontaneously, reduction under anesthesia may be required. Conservative treatment with bracing and physiotherapy remains the standard initial approach in first‐time dislocations. Recurrent patellar dislocation usually requires surgical management for medial patellofemoral ligament reconstruction ± bony procedures or tracheoplasty. Neglected locked patellar dislocation is a rare situation and challenging condition that lacks standardized management strategy and requires a tailored approach for both reduction and soft tissue reconstruction. This technical note describes a combined surgical technique for management of neglected locked patellar dislocation.

## Graphical abstract

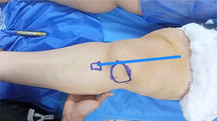

**VIDEO 1:** Annotated procedure video of triple technique for neglected locked patellar dislocation. Video content can be viewed at https://doi.org/10.1002/atn2.70243.

Patellar dislocation is a quite common injury presenting in the emergency department. The medial patellofemoral ligament (MPFL) was found to be torn in first‐time patellar dislocation, occurring in up to 94.7% of cases.^1^ First‐time patellar dislocation may reduce spontaneously, but sometimes, it requires reduction under anesthesia. First‐time dislocations are usually managed conservatively in a brace with physiotherapy.^2^

In neglected dislocations, chronic lateral displacement of the patella leads to adaptive changes including arthrofibrosis, soft‐tissue contracture, quadriceps shortening, and loss of patellar mobility, hindering reduction with standard techniques.^3^, ^4^, ^5^, ^6^, ^7^

This technical note describes a stepwise, reproducible technique for neglected locked patellar dislocations.

## SURGICAL TECHNIQUE

**FIGURE 1:**
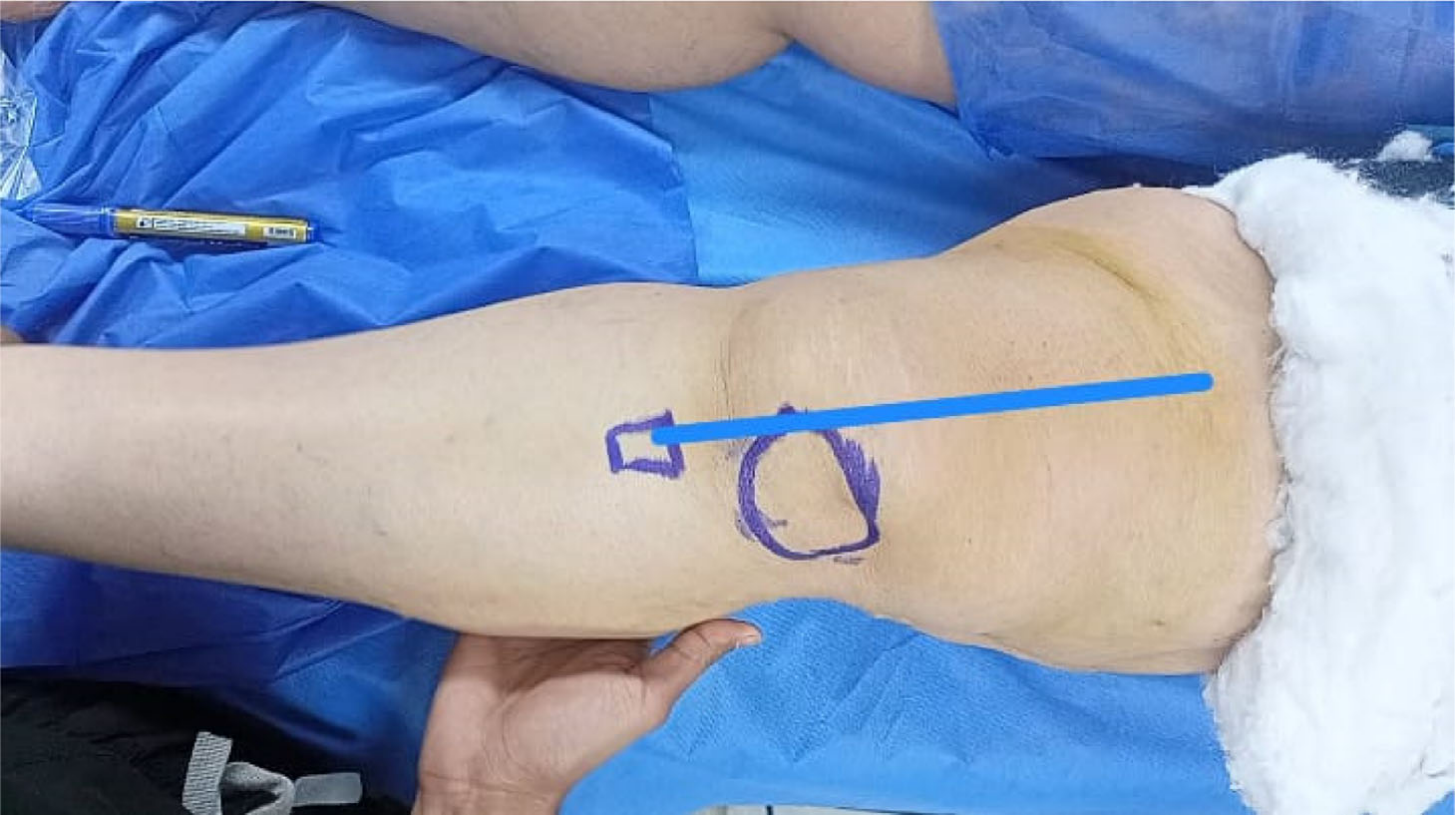
Left knee in the supine position showing a markedly laterally dislocated, locked patella, tibial tuberosity, and marking of the site of skin incision.

**FIGURE 2:**
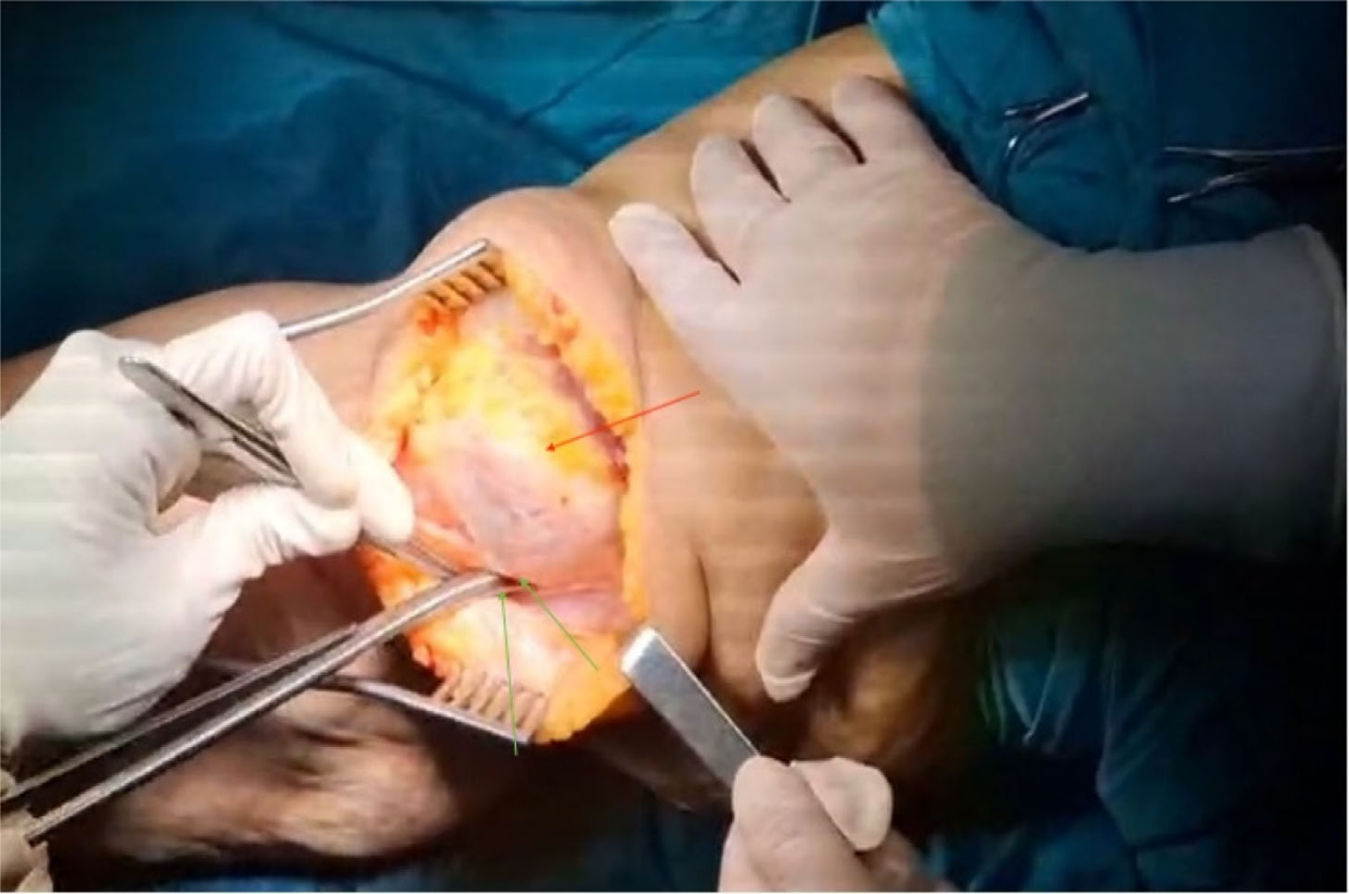
Left knee in the supine position showing lateral release, arthrotomy, and arthrolysis. Green arrows, showing lateral retinacular release and lateral arthrotomy for fat pad excision; red arrow, pointing to the patella.

**FIGURE 3:**
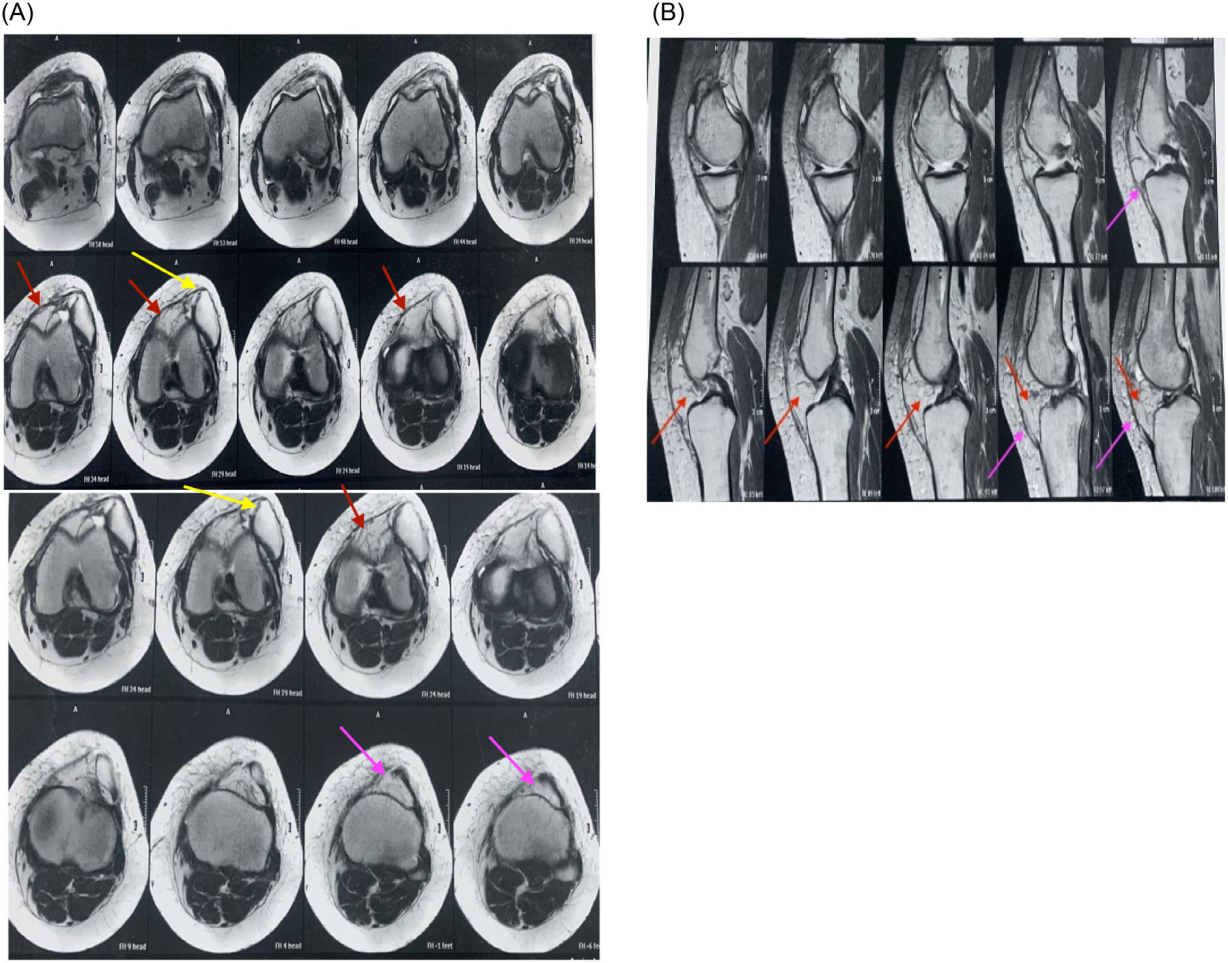
(A) Preoperative axial view MRI of the left knee. Yellow arrows, showing laterally dislocated patella. Red arrows, showing hypertrophied patellar fat pad; pink arrows, showing intact patellar tendon. (B) Preoperative sagittal view MRI of the left knee. Red arrows, showing hypertrophied patellar fat pad; pink arrows, showing intact patellar tendon. (MRI, magnetic resonance imaging.)

**FIGURE 4:**
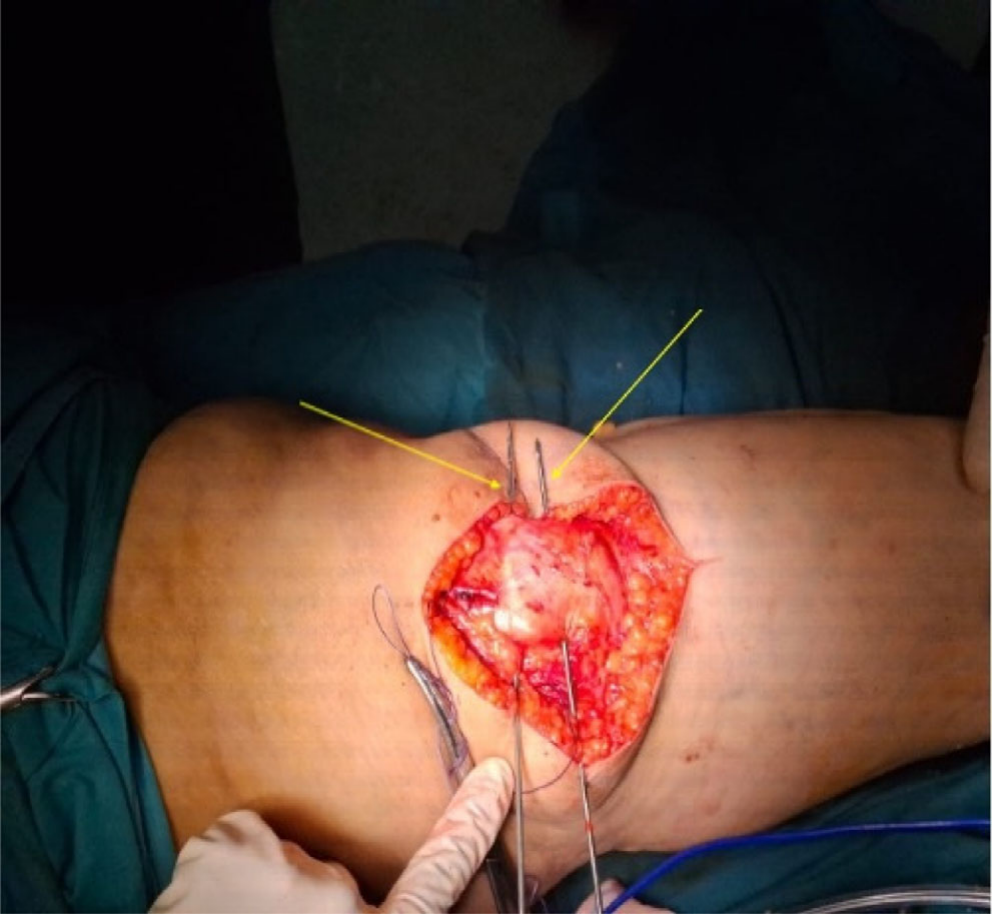
Left knee in the supine position. Yellow arrows, showing 2 thin guide wires passed from medial to lateral through the patella.

**FIGURE 5:**
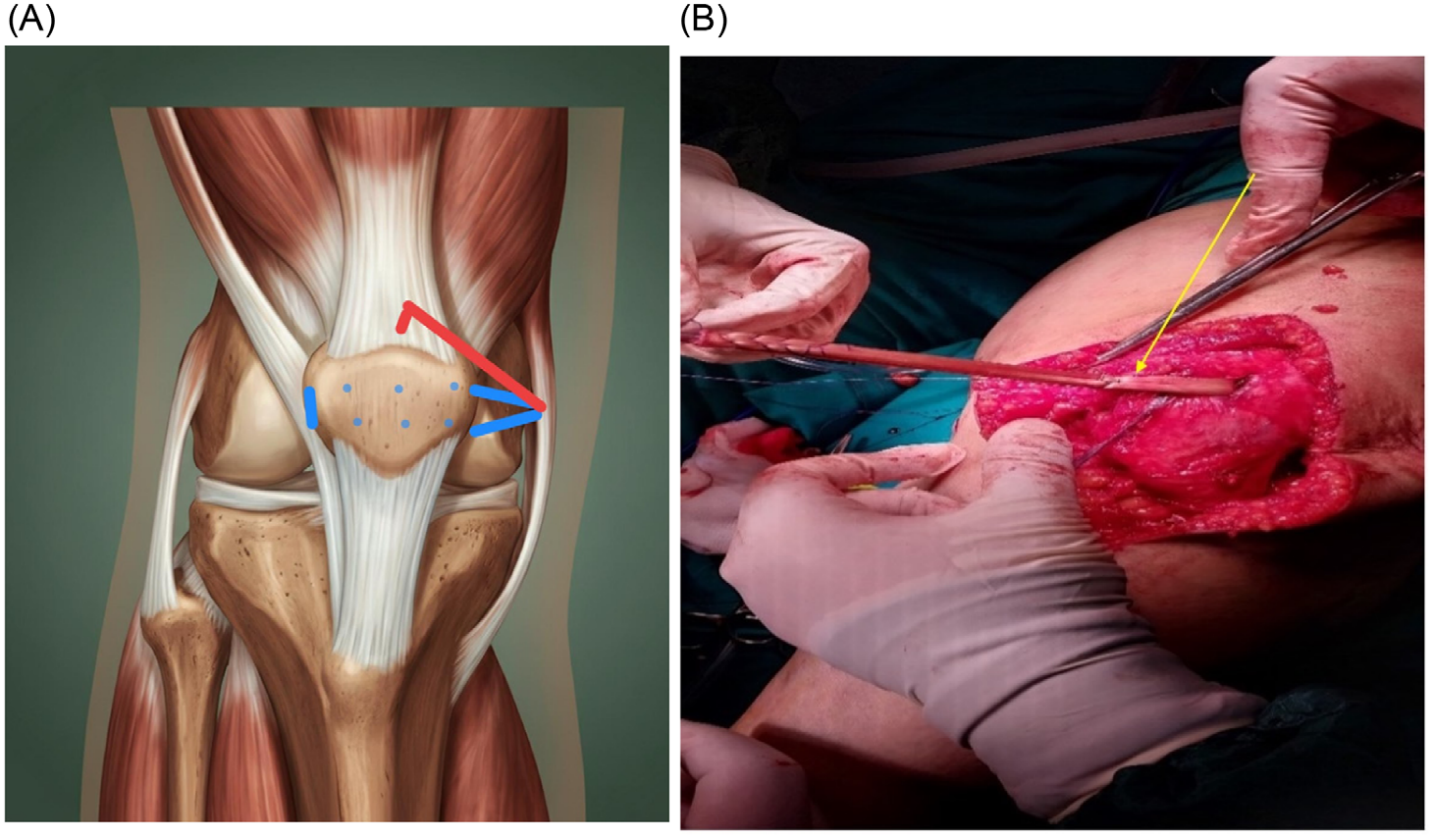
(A) Diagram showing blue lines, illustrating site of FiberTape limbs in the patella with their femoral insertion; gray lines, showing site of patellar tunnels using thin guide wires; and red line, illustrating site of weaved peroneus longus graft loop for MQTFL within the quadriceps tendon and its femoral insertion. (B) Left knee in the supine position showing preparation and stitching of the femoral end of the peroneus longus graft after passage through the quadriceps tendons. Yellow arrow, peroneus longus graft loop for MQTFL weaved through the quadriceps tendon 1 cm proximal to the patella. (MQTFL, medial quadriceps tendon‐femoral ligament.)

**FIGURE 6:**
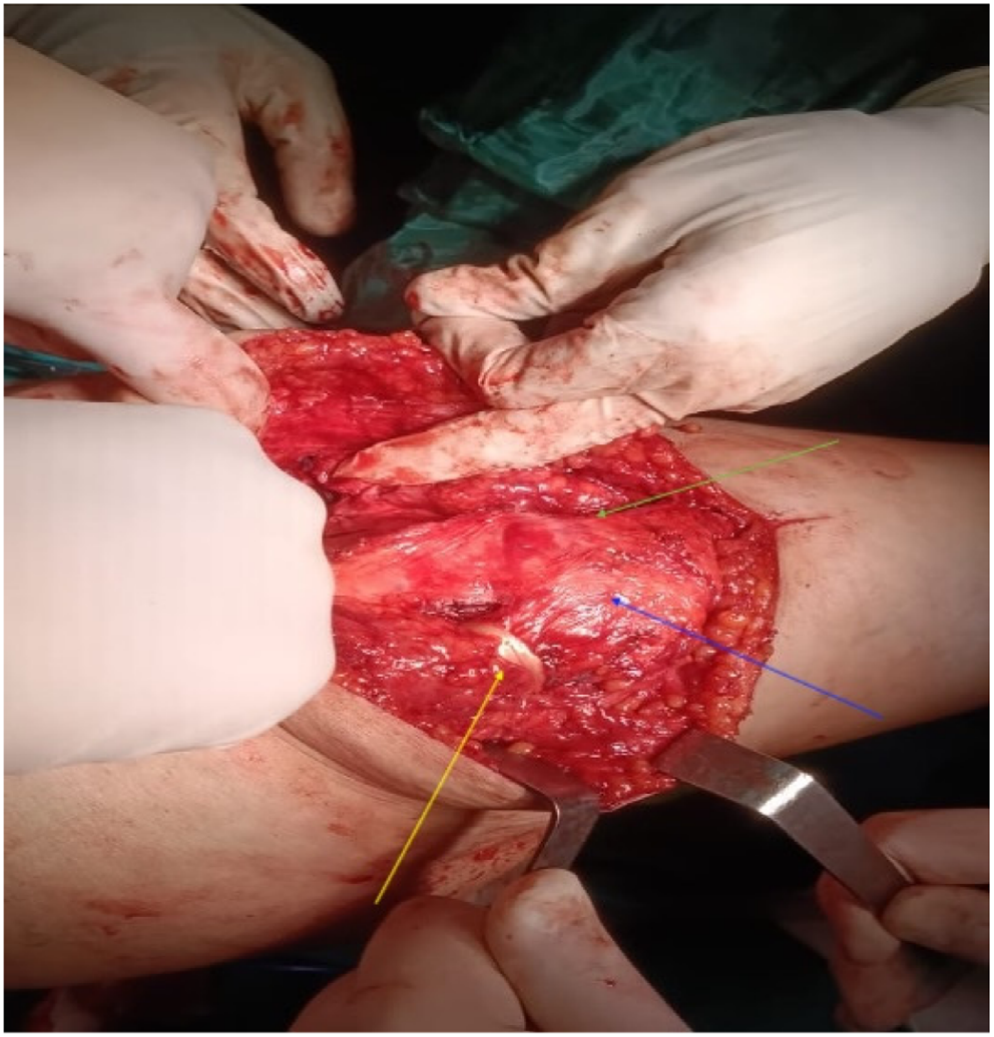
Left knee in the supine position showing patellar relocation in the center of the trochlea after femoral fixation of the reconstructed MQTFL using the peroneus longus graft and the reconstructed MPFL using FiberTape (Arthrex, Naples, FL). Yellow arrow, reconstructed MQTFL; blue arrow, showing patella; green arrow, lateral retinacular release. (MPFL, medial patellofemoral ligament; MQTFL, medial quadriceps tendon‐femoral ligament.)

Each step of the surgical technique is illustrated in Video 1 and Figures 1, 2, 3, 4, 5, 6.

### Patient Setup

The patient is positioned supine after spinal anesthesia draped. Patella and tibial tuberosity landmarks are marked as shown in Figure 1. Examination confirms fixed, irreducible lateral patellar dislocation, limited knee flexion, and range of motion (ROM).

### Graft Preparation

Peroneus longus (PL) autograft was used for medial quadriceps tendon‐femoral ligament (MQTFL) reconstruction because of its larger length and diameter compared with hamstring tendons. A skin incision was made posterior to the lateral malleolus, the PL tendon was identified, and distal tenodesis of the PL tendon to the peroneus brevis was performed before harvesting to maintain proper tendon tension. Then, graft is harvested, prepared, and Whip‐stitched on both ends using FiberWire No. 2 suture (Arthrex, Naples, FL).

### Skin Incision

Midline skin incision is made starting at the midpoint of the thigh, directed distally to the tibial tuberosity. Care is taken to avoid shifting the incision laterally toward the patella (Figure 1). Subcutaneous dissection is carried out toward the vastus lateralis, yielding a generous subcutaneous flap to avoid postoperative wound closure complications.

### The First Technique

Knee arthrotomy is performed at the lateral border of the patella, releasing the patella from the lateral retinaculum (Figure 2). Careful dissection is carried out distally toward the tibial tuberosity along the lateral border of the laterally shifted patellar tendon to avoid injury to the tendon.

Proximally, the vastus lateralis is released at the rectus femoris. Then, the patella is externally rotated to expose the undersurface and excise the hypertrophied fibrous infrapatellar fat pad, allowing relocation of the patella into the trochlear groove (Figure 3).

### The Second Technique

#### Synthetic MPFL Reconstruction

Two parallel guide wires are passed medial to lateral with 1 cm interval in‐between centrally in the patella confirmed under image intensifier to avoid intra‐articular or bony breach (Figure 4).

FiberTape (Arthrex, Naples, FL) is shuttled through the patella in a loop fashion with 1 limb passing via the proximal wire from medial to lateral; then, the same limb is passed through in the distal wire from lateral to medial, so the FiberTape (Arthrex, Naples, FL) is securing the patella with the 2 limbs on the medial side.

### The Third Technique

#### MQTFL Reconstruction

A proximal 1‐cm incision is made at the junction between the medial and central third of the distal quadriceps tendon attachment at the superior border of the patella; then, a distal 1 cm partial‐thickness incision is made at the superior‐medial border of the patella through layers 1 and 2 without penetrating layer 3. A curved clamp is used to create a passage between the 2 incisions without penetrating the capsule. The PL graft is passed through the 2 incisions using the clamp, creating a loop (Figure 5A,B). The 2 limbs of the graft are stitched proximally near the distal incision using FiberWire No. 2 suture (Arthrex, Naples, FL). The loop is secured to the quadriceps tendon using FiberWire No. 2 suture (Arthrex, Naples, FL).

### Femoral Tunnel

A 2‐cm incision is created between the medial epicondyle and the adductor tubercle. The femoral Schöttle point, proximal and posterior to the medial epicondyle, is identified using an image intensifier, and then, a guide wire is inserted. The PL graft is looped around the guide wire, and the knee is brought into ROM to confirm that the graft is isometric.

The guide wire is advanced until it passes through the skin of the lateral side of the femur.

A small skin incision is made at the site of the guide wire exit, and subcutaneous dissection is continued toward the femur lateral cortex.

A reamer is used to create the femoral tunnel according to the graft diameter; then, a No. 2 Vicryl suture (Ethicon, Johnson & Johnson, Somerville, NJ) is passed via the guide wire from medial to lateral.

### Passage of the Reconstruction Components

A clamp is used to pass both the graft and the FiberTape (Arthrex, Naples, FL) from the incision at the femoral tunnel to the incision at the superomedial border of the patella without penetrating layer 3.

The Vicryl (Ethicon, Johnson & Johnson, Somerville, NJ) suture is used to shuttle both the graft limbs and the FiberTape (Arthrex, Naples, FL) limbs through the femoral tunnel until the suture limbs stitched to the graft and the limbs of the FiberTape (Arthrex, Naples, FL) are retrieved from the lateral skin.

At this stage, both reconstruction components are fixed at the same isometric Schöttle point with 2 different lines of pull (Figure 5A).

### Fixation of the Reconstruction Components

The knee is brought into 30° of flexion, and any slack of the graft or the tape is removed. The patella is manually reduced into the trochlear groove. The graft is then tensioned and secured in the tunnel with a PEEK Interference Screw, 8 **× **23 mm (Arthrex, Naples, FL).

The 2 limbs of the FiberTape (Arthrex, Naples, FL) are secured to the lateral femoral cortex with half hitches using TightRope Attachable Button System ABS (Arthrex, Naples, FL), yielding a stable final construct (Figure 6).

Final assessment confirms centralized patellar tracking, stability, and knee ROM, as well as appropriate tension of both reconstruction components and absence of excessive medial constraint.

### Rehabilitation

A knee immobilizer is applied for 3 weeks. Knee flexion is initiated to 30° in the third postoperative week and is subsequently increased by 30° each week. Weight‐bearing is restricted for the first 2 weeks. Static quadriceps strengthening exercises are initiated early in the postoperative period.

Key technical pearls and pitfalls of this technique are summarized in Table 1.

**TABLE 1:** Pearls and Pitfalls

Pearls	Pitfalls
‐ Make the skin incision in the midline rather than over the dislocated patella ‐ Develop adequate subcutaneous flap to reduce the risk of skin necrosis ‐ Perform aggressive excision of hypertrophied infrapatellar fat pad to facilitate patellar reduction ‐ Perform lateral release carefully to avoid iatrogenic injury to the patellar tendon ‐ Confirm graft isometry and patellar tracking throughout full range of motion before final fixation	‐ Penetration of layer 3 during graft and FiberTape passage ‐ Breaching the lateral femoral cortex during tunnel reaming, compromising button fixation ‐ Incorrect graft length leading to laxity if excessively long ‐ Inadequate control during graft passage may result in malposition

### Ethical Approval

A written informed consent was obtained from all participants.

## DISCUSSION

Management of neglected patellar dislocation is rare and presents challenges distinct from recurrent patellar instability, particularly in long‐standing cases with fixed deformity and soft‐tissue contracture. Although medial patellar stabilization procedures have shown favorable outcomes in recurrent instability, their application to neglected locked dislocations requires additional considerations.^8^

The MPFL and the MQTFL are well‐established components of the medial patellar restraint complex.^9^ Together, they provide static stabilization of the patella throughout knee flexion and extension, forming the biomechanical basis for medial restraint reconstruction.^2^, ^4^

Multiple techniques for MQTFL reconstruction have been described, with the principal advantage of avoiding large patellar tunnels and thereby reducing the risk of patellar fracture.^3^, ^10^, ^11^, ^12^ In parallel, synthetic MPFL reconstruction using suture tape constructs has been reported as a reliable and reproducible option, typically fixed at the anatomic femoral footprint described at Schöttle point.^13^, ^14^, ^15^ Biomechanical studies have shown that suture tape constructs with knotless fixation may provide superior load‐to‐failure characteristics compared with soft‐tissue autografts.^5^, ^9^

In neglected cases, extensive lateral soft‐tissue contracture is common, making lateral release and patellar mobilization mandatory before medial reconstruction can be effective. Lateral release was preferred over Z‐lengthening techniques as it allows direct decompression of the contracted lateral retinaculum, facilitates immediate patellar reduction, and avoids additional tendon lengthening or quadriceps muscle weakness that may increase surgical complexity and morbidity. This step must be performed meticulously with adequate subcutaneous flap development to allow safe patellar mobilization and subsequent medial reconstruction. In addition, long‐term disuse of the patella and extensor mechanism frequently results in osteopenia, increasing the risk of patellar fracture when conventional tunnel‐based techniques are used.^15^

In this technique, large patellar tunnels are avoided through the use of small‐diameter guide wires and suture tape fixation. The MQTFL reconstruction allows redistribution of stabilizing forces through the quadriceps mechanism, reducing stress concentration on the patella. Fixation of both the synthetic MPFL and the MQTFL graft at a single anatomic femoral footprint provides 2 complementary lines of pull while maintaining isometry throughout knee ROM.

This combined approach offers advantages, including enhanced medial stabilization, load sharing between reconstruction components, and reduction of fracture risk in compromised bone. The limitations include potential risks of wound complications and donor‐site morbidity related to autograft harvest.

The advantages and disadvantages of this technique are summarized in Table 2.

**TABLE 2:** Advantages and Disadvantages

Advantages	Disadvantages
‐ The open approach allows adequate lateral release and excision of adhesions, facilitating reduction of the patella ‐ Provides dual lines of pull over the patella directed toward a single isometric Schöttle point ‐ Uses 2 fixation methods, enhancing construct stability and patellar reduction ‐ Synthetic MPFL reconstruction avoids large patellar tunnels, reducing the risk of iatrogenic fracture ‐ MQTFL reconstruction provides a dynamic stabilizing mechanism through the quadriceps tendon	‐ Open technique may be associated with increased risk of scarring and infection ‐ Extensive lateral release may increase the risk of patellar osteonecrosis ‐ Requires careful graft tensioning to avoid overconstraint ‐ Donor‐site morbidity related to autograft harvest

MPFL, medial patellofemoral ligament; MQTFL, medial quadriceps tendon‐femoral ligament.

## DISCLOSURES

The authors (H.A.A.G., M.K.E.G., A.S.M.E.H., A.H.K.) declare that they have no known competing financial interests or personal relationships that could have appeared to influence the work reported in this article.
